# Primary stratification and identification of suspected Corona virus disease 2019 (COVID-19) from clinical perspective by a simple scoring proposal

**DOI:** 10.1186/s40779-020-00246-8

**Published:** 2020-04-04

**Authors:** Ting-Ting Zhou, Feng-Xian Wei

**Affiliations:** 1 Department of Respiratory Medicine, Lanzhou University Second Hospital, Lanzhou, 730030 Gansu China; 2 Lanzhou University Second Clinical Medical College, Lanzhou University, Cuiyingmen 82, Chengguan District, Lanzhou, 730030 Gansu China

**Keywords:** COVID-19, Suspected cases, Primary screening, CT imaging, Blood test

## Abstract

In this Commentary, we would like to comment on the article titled “A rapid advice guideline for the diagnosis and treatment of 2019 novel coronavirus (2019-nCoV) infected pneumonia (standard version)” as a featured article in *Military Medical Research*. In the guideline, except for “confirmed cases”, “suspected cases”, “close contact” and “suspicious exposure” were defined by clinical perspective based on epidemiological risk, clinical symptoms and auxiliary examination. Combined with our experience, we introduced a simple scoring proposal additionally based on not only CT imaging as strongly recommended by the guideline but also blood routine test, especially for primary screening of such patients in the out-patient department.

**Dear Editor,**

The featured article “A rapid advice guideline for the diagnosis and treatment of 2019 novel coronavirus (2019-nCoV) infected pneumonia (standard version)” was the first guideline in English version for the management of Corona Virus Disease 2019 (COVID-19) in China [1] as we known, and also the Chinese experts proposed “advice guideline for the diagnosis and treatment of 2019 novel coronavirus (2019-nCoV) infected pneumonia” in Chinese version. The advice guideline has been updated to the seventh version [2] based on clinical evidence, experience and the requirement of epidemic prevention. Certainly, clinical practice has proved the effectiveness of these guidelines, and the incidence of COVID-19 has significantly reduced in most of the areas in China. While, as reported by media, the situation outside of China, especially in Italy, Iran, Spain, Republic of Korea, France, German, United States of America and other countries seem to be changed. By 18 March 2020, the total incidence of COVID-19 is estimated by more than 191,000 cases [3]. Respiratory droplets and close contact are still the main routes of transmission [1, 4].

Based on our experience, we would like to comment and add additional information of diagnosis and primary screening by the following 2 topics:

Firstly, early diagnosis and isolation of infections are essential for preventing further spread, which are also the most important parts of the pandemic management [4]. For the diagnosis, nucleic acid detection as stated in the guideline is still served as a “gold standard”, and most time well-collected throat swab specimen is enough for diagnosis. While, it was reported that the false negative rate was sometimes to be relatively high to as 50% in a single detection [5], mainly because that the specimen from the upper other than the lower respiratory tract usually contained less amount of severe acute respiratory syndrome coronavirus (SARS-CoV-2), especially for mild type and recessive patients [6]. Hence, multiple sites and time points of tests are required, and even bronchoalveolar lavage fluid (BALF) is required for “highly suspected patients”. Besides, test for newly developed specific serum antibody can provide more accurate results, and IgM antibody which appeared to be positive within 3 to 5 days after onset in 3 to 5 days and IgG antibody increased over 4 times than that in the acute phase [2]. With the above positive finding, the patients can be defined as “confirmed cases”.

Secondly, stratification and identification of potentially infected patients are also very important in the clinic, thereby further management should be performed by individualization. For the diagnosis of “suspected patients”, “close contact” and “suspicious exposure”, the guideline and multidisciplinary experts focused any of epidemiological risk such as “a history of travel to or residence in Wuhan city, China or other cities with continuous transmission of local cases in the last 14 days before symptom onset; contact with patients with fever or respiratory symptoms from Wuhan city, China or other cities with continuous transmission of local cases in the last 14 days before symptom onset; or epidemiologically connected to COVID-19 infections or clustered onsets” [1, 2]. However, in the clinical practice, from the first onset case to now, the epidemiological risk evaluation seems to be harder and harder for the doctors, as more and more patients in the out-patient department and emergency department cannot clearly state their contact with other potential infections. Additionally, attention need to be focused on such patients. After collecting the data of patients from our hospital and combining with our clinical experience [8], we proposed a simple stratification process for COVID-19 based on all 4 items: epidemiological risk assessment, clinical symptom assessment, blood routine assessment and chest CT assessment; As shown in Fig. 1. The former 2 items can complete by consultation, and the later 2 items can be easily conducted in most hospitals. Specifically, CT imaging is always the important reference for clinical diagnosis [1, 7], meanwhile more and more clinical studies demonstrate the value of blood routine parameters for differential diagnosis for COVID-19 [8, 9]. As strongly recommended in this guideline [1], typical CT imaging can be used for the stage diagnosis, and with nucleic acid detection as a reference, the sensitivity and specificity of chest CT imaging were calculated to be 97 and 25%, respectively [5]. Besides, blood routine parameters actually can provide important information, for the differential diagnosis from other community acquired pneumonia [8, 9], as well as for the severity of disease since the blood routine parameters are significantly different between no-severe and severe type of COVID-19 patients as reported in the large scale study by Zhong Nan-Shan et al [4]. Also, with nucleic acid detection as a reference, when blood routine test parameters were presented as normal, reduced or increased with normal limits, the sensitivity and specificity were calculated to be 89 and 34%, respectively [9], and multiple parameters analysis in blood routine were helpful to further increase the specificity [8]. Meanwhile, when they were presented as optimal cut-off value, the specificity was calculated to be significantly increased to nearly 80% [8]. Taking together all the above reported data, a combination of blood routine test and CT imaging would significantly increase the primary screening efficacy. Combined with our experience and current diagnostic process in this guideline, we proposed a scoring proposal including additionally specific parameters in the blood routine test and typical manifestations in the CT imaging to increase the clinical feasibility. After application in the clinic, it was simple and rapid for primary screening, and was easy to follow for both doctors and nurses. Our study aiming at the accurate data on its primary screening efficacy is still ongoing.

**Fig. 1:**
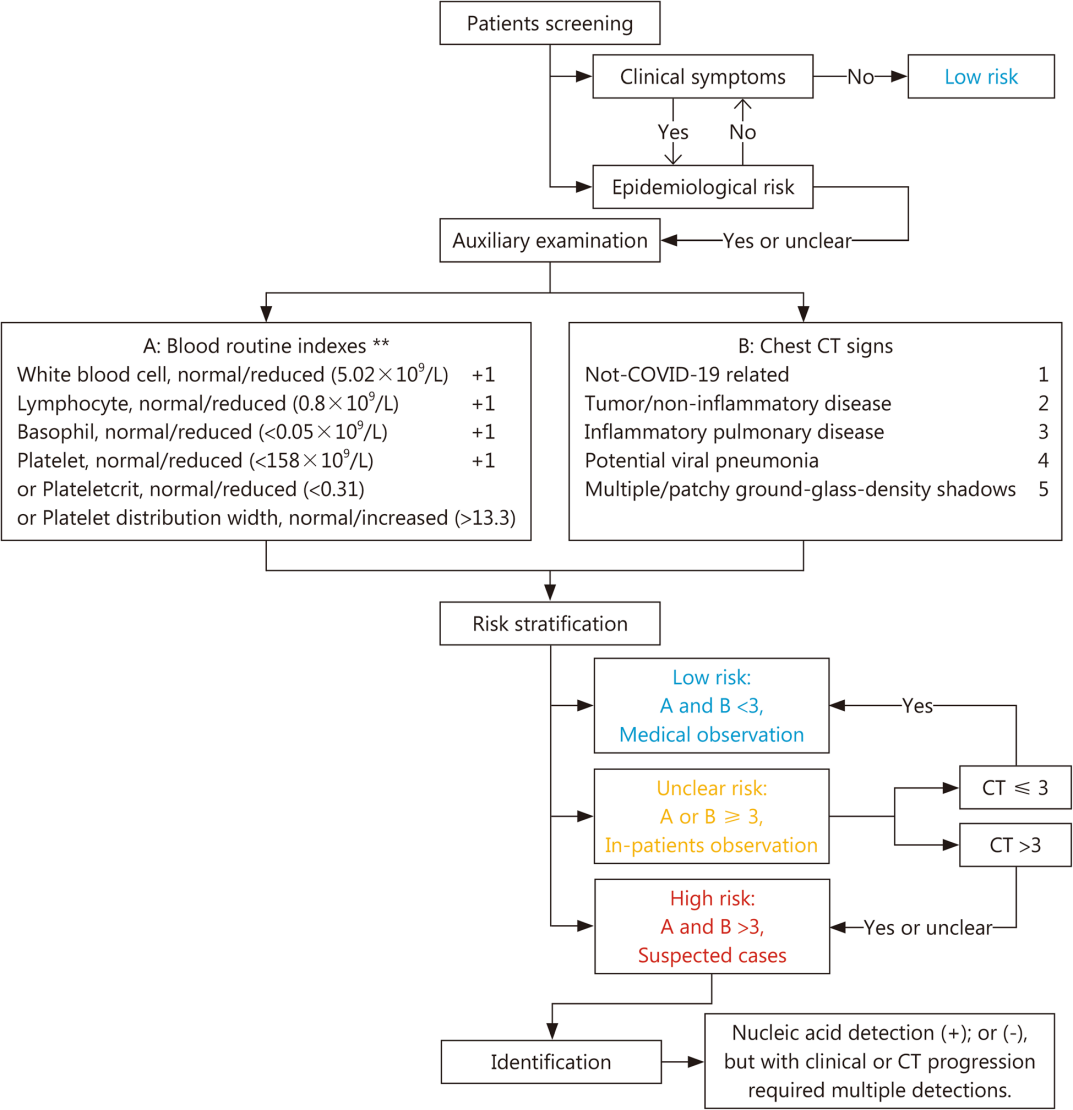
Flow chart of simple stratification and identification of COVID-19 **. Blood routine parameters were presented as normal, reduced or increased with normal limits, and also as optimal cut-off value based on our retrospective analysis results. COVID-19. Corona Virus Disease 2019

Clearly, although the final diagnosis of COVID-19 requires nucleic acid, Immunoglobulin M (IgM) and/or Immunoglobulin G (IgG) antibody tests, this proposal can be applied to a large range of COVID-19-related population in routinely equipped hospitals for primary screening of high-risk patients. Based on our experience, this would be useful for the simple and primary stratification and identification, especially in undeveloped or developing countries and areas lacking experience and even lacking sufficient specialist physicians. Meanwhile, further clinical data is still warranted to validate the experience and its application.

## Data Availability

Data sharing is not applicable to this article as no datasets were generated or analyzed during the current study.

## Abbreviations

2019-nCoV: 2019 Novel coronavirus
BALF: Bronchoalveolar lavage fluid
COVID-19: Corona virus disease 2019
IgM: Immunoglobulin M
IgG: Immunoglobulin G
SARS-CoV-2: Severe acute respiratory syndrome coronavirus
